# Insights into the connection between pathogen water eDNA and disease progression in zebrafish infected with *Vibrio anguillarum*

**DOI:** 10.1016/j.cirep.2024.200160

**Published:** 2024-07-14

**Authors:** Cyril Henard, Manuel Blonç, Hanxi Li, Yajiao Duan, Louise von Gersdorff Jørgensen

**Affiliations:** a Department of Veterinary and Animal Sciences, University of Copenhagen, Stigbøjlen 7, Frederiksberg C, 1870, Denmark; b Department of Cell Biology, Physiology and Immunology, Universitat Autònoma de Barcelona, Spain; c Department of Health Technology, Technical University of Denmark, Kongens Lyngby, Denmark

**Keywords:** Environmental DNA, Zebrafish, *Vibrio anguillarum*, Immune response, qPCR, Stress

## Abstract

The host-pathogen interactions are tripartite and include the host, the pathogen, and the environment. For a long time, attention has mainly been addressed to the host's immune mechanisms and the pathogen's virulence factors while the environmental factor has received less interest. About a decade ago, studies on environmental DNA (eDNA) started to emerge as this approach is versatile for numerous research fields and interest is persistently growing. In the present study, the relationship between disease progression and pathogen water environmental DNA level was investigated. To perform this investigation, an infection model was setup using zebrafish and the gram-negative bacteria *Vibrio anguillarum*. Two different fish densities and bacterial concentrations were used to determine different patterns of pathogen eDNA in the fish tanks. Water sampling to collect eDNA was conducted alongside with observation of the fish Clinical Signs, mortalities, and immune responses. Water eDNA relative abundance of the pathogen could be determined directly after fish challenge, when Clinical Signs occurred, and when mortality started. Moreover, immune-relevant gene expression in zebrafish gills showed that *il-1β, il-8, il-10, nf-κb* and *saa* correlated to the pathogen eDNA signal, depending on fish density and bacterial concentration. In conclusion, this study demonstrated that water eDNA is a promising non-invasive tool to state disease progression and immune response in zebrafish infected with *V. anguillarum*.

## Highlights

• Water environmental DNA (eDNA) is a promising tool to follow disease progression in fish.
• Water eDNA profile of *V. anguillarum* correlates to immune gene expression of zebrafish gills.
• Crowding of zebrafish modulate the immune gene expression against *V. anguillarum*.
• The *nf-κb* gene expression in zebrafish gills was conserved among all experimental conditions.

## Introduction

In the environment, DNA may have various origins such as active and dormant cells, cysts, eggs, faeces, dead organisms and shedding from organisms such as mucus [1]. The term environmental DNA (eDNA) defines the genetic material present in the environment that originates from living and dead organisms and that can be collected, stored, purified, amplified, sequenced and determined [2]. In 1987, Andrew Ogram was the first to use the term “environmental DNA” in the title of the protocol diagram he used in a study that aimed to collect microbial DNA from sediment [3]. In 2012, a study describes eDNA as: “DNA that can be extracted from environmental samples (such as soil, water or air), without first isolating any target organisms” [4]. Since then, eDNA applications such as eDNA metabarcoding have expanded to various research fields including air quality monitoring, ancient ecosystems, community pollution response, plant-pollinator interactions, detection of invasive species, diet analysis, as reviewed by Ruppert et al. in 2019 [2]. In aquatic environments, the distribution of eDNA in the water column is complex and the former lack of knowledge regarding the factors which contribute to this intricacy conducted to many investigations [5]. Nonetheless, several studies use eDNA as a tool to detect the presence of pathogens [6,7], estimate invasive species population and distribution [8], evaluate the impact of aquaculture on the environment [9,10] and perform non-invasive sampling for genotyping [11]. Despite a growing interest in this methodology, eDNA relative abundance of fish pathogens have not being associated with Clinical Signs and disease progression. In previous studies, the host-pathogen interactions between *V. anguillarum*, causing vibriosis in fish [12], and the zebrafish (*Danio rerio*) were described. The zebrafish immune response after bath vaccination has been described by Zhang et al. [13] and disease progression using imaging was performed by Schmidt et al. [14]. In the present study, a waterborne infection of zebrafish with different concentrations of *V. anguillarum* was performed to pinpoint water eDNA relative abundance during a lethal and a non-lethal exposure and the correlation to Clinical Signs and mortality. In addition, we performed this infection experiment with two fish densities to evaluate if a high host density would impact the fish immune response, the progression of disease and the water eDNA level of *V. anguillarum*. The selected immune genes were investigated according to their relevance in inflammatory process (*il-1β, il-8, nf-κb, tnf-α*), immune regulation (*il-10, tgf-β*), acute response (*saa*) and host defence complex (*c3, igm*). Finally, a correlation between pathogen water eDNA and the fish immune response was conducted to highlight the potential of the approach as a non-invasive bioindicator of disease state.

## Material and method

### Fish

Adult wild type zebrafish (AB) were reared in a recirculated system (Aquaschwartz, Germany) at 27 °C with a pH of 7.5 and conductivity of 650 ms with a light/dark cycle of 14/10 h. The fish were fed with both live prey (Sep-Art Artemia Cysts, Ocean Nutrition™) and pelleted dry feed (ZM300 Fish Food, England) three times per day. The fish were randomly allocated in the experimental tanks 24 h before the challenge for acclimation.

### Bacteria

*V. anguillarum* (6018/1) was cultivated in Luria-Bertani broth at 25 °C, 130 rpm. Prior to infection, the bacterial culture was centrifuged for 10 min at 2500 g. The pellet was resuspended in PBS 1X (Calbiochem®) twice before being resuspended with filtered (0.2 µm) zebrafish facility water. The OD_600_ was measured with a spectrophotometer (SmartSpec™ 3000, Bio-Rad) and the bacterial culture was used for infection at OD_600_ 1.2 corresponding to the exponential growth phase. The estimation of colony forming unit (CFU) concentration was assessed with bacterial numbering on blood agar plates.

### Fish densities and challenge

Twelve tanks containing adult zebrafish (*n* = 20) were set up for the experimental challenge. Half of the tanks were filled with 4 L of facility water (5 fish/L, control fish density) and the other half were filled with 2 L (10 fish/L, crowded fish density) resulting in crowding which induces mild stress and significantly elevated levels of cortisol in fish as described in a previous study [15]. The waterborne challenge method employed in this study originated from the work of Schmidt et al. (2017) with minor modification [14]. In a dedicated sterile beaker (500 mL), the fish were infected by waterborne challenge for 1 min with either high concentration (3.14 × 10^8^ CFU/mL), low concentration (2.15 × 10^5^ CFU/mL) of bacterial culture or mock infected (facility water) in duplicates for the two fish densities. After the waterborne challenge, the fish were maintained in the air few seconds to remove the carryover of the bacterial solution and returned to their tanks. Following the bacterial exposure, the fish mortalities were recorded until seven days post-infection. The moribund fish (unable to control balance) were euthanised with an overdose (400 mg/L) of MS222 (tricaine methane sulphonate, Sigma-Aldrich) and included as mortalities. The four experimentally infected fish groups were defined as low bacterial concentration with 5 fish/L (low), high bacterial concentration with 5 fish/L (high), low bacterial concentration with 10 fish/L (low-crowded), high bacterial concentration with 10 fish/L (high-crowded). In addition, two uninfected control groups for each fish densities were used as control for either non-crowded (5 fish/L) and crowded (10 fish/L) fish densities.

### Sampling for qPCR

#### Tissue sampling and Clinical Signs

Fish (*n* = 4 per tank) gills were sampled for gene expression at 5, 24, 72, 120 and 168 h post infection (HPI) and placed in RNA*later*® (Sigma-Aldrich) at 4 °C for 24 h, then −20 °C until RNA purification. The sampled fish were euthanised with an overdose (400 mg/L) of MS222 and examined under binocular microscope for Clinical Signs. The control groups (mock-infected) were used as reference to evaluate severity of Clinical Signs of infected groups and as time point controls for qPCR. The observed Clinical Sings characteristic of vibriosis [12] were assigned to none (identical as control group), mild (light inflammation, minor reddish area around pectoral and pelvic fins, and abdomen area) and severe (haemorrhages, splenomegaly, necrotic tissue).

#### Water sampling

Water samples (500 mL per tank) were collected at 5, 24, 48, 72, 96, 120, 168, 214, 216 HPI and kept overnight at 4 °C until processing. The sampled water was replaced with facility water. Water samples were filtered through a 0.45 µm membrane (Cellulose acetate, ADVANTEC®) until the whole sample had been processed or until the filter had clogged. Directly following filtration, the filters were dry frozen at −20 °C until eDNA purification.

### Nucleic acid purification

#### Zebrafish gills RNA purification

Total RNA from zebrafish gills was purified with GenElute™ Mammalian Total RNA Miniprep Kit (SIGMA®). Gills were lysed with a sonicator (Sonic Dismembrator Model 300, Artek) with alternating sonication and cooling in five seconds intervals. The tubes containing the organs were placed in a beaker containing ice and water to protect the samples from heat. RNA purification was performed following manufacturer's instructions with minor changes. For the gill samples, a proteinase K digestion (55 °C, 400 rpm, 10 min) was implemented. After RNA purification, samples were treated to remove DNA as follows: For each sample, 2.5 µL of DNase I Amplification Grade (Sigma-Aldrich) and 2.5 µL of 10x Reaction Buffer (Sigma-Aldrich) were added. The samples were incubated at 37 °C for 30 min for DNase treatment. To stop the DNase treatment, 2.5 µL of Stop solution (Sigma-Aldrich) was added and samples were incubated at 65 °C for 10 min. RNA concentration was assessed with NanoDrop 2000 (Thermo Scientific). RNA integrity was assessed with electrophoresis using an agarose gel (1.5 %) containing ethidium bromide. The samples were loaded with RNA Sample Loading Buffer (Sigma-Aldrich). The purified RNA samples were stored at −80 °C until subsequent cDNA synthesis.

#### Water eDNA purification

The eDNA samples (whole filters) were purified with DNeasy® PowerWater® Kit (Qiagen), following manufacturer's instructions. The concentration was assessed with NanoDrop 2000 (Thermo Scientific). The purified samples were stored at −20 °C until qPCR analysis.

#### Zebrafish gills cDNA synthesis

The cDNA synthesis was performed with TaqMan® Reverse Transcription kit (Applied Biosystems®). The reaction volume per sample was composed of 10 µL of mastermix (1.16 µL H_2_0, 2 µL 10X RT Buffer, 0.44 µL MgCl_2_ 250 mM, 4 µL dNTP Mix, 0.4 µL Primer, 1 µL RNase inhibitor, 1 µL MultiScribe reverse transcriptase) and 10 µL of template (600 ng of RNA per sample and variable volume of H_2_0). The reaction was conducted with T100™ Thermal Cycler (Bio-Rad) with the following program: 25 °C for 10 min, 37 °C for 60 min, 95 °C for 5 min. Samples were diluted with 180 µL of DNase, RNase free H_2_O. Samples were store at 4 °C for short term and −20 °C for long term.

### qPCR

#### Zebrafish gills

Gene expression analyses of zebrafish gills has been conducted on an AriaMx Real-Time PCR System (Agilent). A list of primers and probes used in this study can be found in Table 1. The final reaction volume for gene expression analysis was 12.5 µL and includes, 6.25 µL Brillant III Ultra-Fast QPCR Master Mix (Agilent Technologies), 1 µL of primer mix (primers: 10 µM, probe: 5 µM), 2.75 µL of H_2_O and 2.5 µL of cDNA template. The qPCR assays used in the present study exhibited efficiencies within 100 ± 5 % and data were analysed using a simplified 2^-ΔΔCt^ method [16]. Thermal conditions consisted of a pre-denaturing step for 3 min at 95 °C followed by 40 cycles with denaturing for 5 s at 95 °C and elongation for 15 s at 60 °C. The eDNA analysis was done with the same equipment, although a different protocol. The final volume for analysis was 30 µL, which included, 15 µL TaqMan™ Environmental Master Mix 2.0 (Applied Biosystems™), 3 µL of primer mix (primers: 10 µM, probe: 5 µM), 7 µL of H_2_O and 5 µL of DNA template. The thermal profile used consisted of a pre-denaturing step for 10 min at 95 °C followed by 45 cycles with denaturing for 15 s at 95 °C and elongation for 60 s at 60 °C.

**Table 1:** Primers used for eDNA and gene expression analyse. F: forward. R: reverse. P: probe.

Gene	Species	GenBank Accession number	Sequence 5′−3′	Product size (bp)
β*-actin*	*Danio rerio*	BC154531	F: CCATCCTTCTTGGGTATGGA	91
			R: ACAGGTCCTTACGGATGTCG	
			P: TGCGGTATCCACGAGACCACC	
*c3*	*Danio rerio*	NM_131,242 NM_131,243 NM_001037236	F: TGCTGTTCTTCTCTCCTCAGC	88
			R: WGAKGAACCCACTCTCAGCA	
			P: CTCACACTGTGTGACCCGCTAT	
*il-8*	*Danio rerio*	XM_001342570	F: GATCTGTCTGGACCCCTCTG	79
			R: GGGCATTCATGGTTTTCTGT	
			P: CCATGGGTTAAGAAGATCATTGATAGG	
*elf1*α	*Danio rerio*	AY422992	F: GAACGACCCACCCATGGAGG	158
			R: TGATGACCTGAGCGTTGAAG	
			P: GAACGACCCACCCATGGAGG	
*igm*	*Danio rerio*	AY643753	F: TGCAGTTCTGGTTCTGATGG	122
			R: TGCACAAAATCGCTCAAATC	
			P: AATCACCCTCGGCTGCTTGG	
*il-10*	*Danio rerio*	BC163038	F: CTTGCCAAAATCCCTTTGAA	92
			R: ATCAAGCTCCCCCATAGCTT	
			P: TGAAAAGATGAAGGAAAAGGGGG	
*il-1β*	*Danio rerio*	BC098597	F: CGCTCCACATCTCGTACTCA	166
			R: ATACGCGGTGCTGATAAACC	
			P: GAAGGAGACCGGCAGCTCCA	
*nf-*κ*b*	*Danio rerio*	BC122885	F: CAGGATTTGGACAAYGAGGT	76
			R: CCAGTTCCCTCCAAGGTACA	
			P: TGAGCAAGCTGTGTGGGATTCT	
*rpl13a*	*Danio rerio*	NM_212,784	F: TCCCAGCTGCTCTCAAGATT	87
			R: ACTTCCAGCCAACTTCATGG	
			P: CACACGCAAATTTGCCCTGC	
*saa*	*Danio rerio*	BC081487	F: CTTGCTGTGCTGGTGATGTT	129
			R: CTTCCAATTGGCCTCTTTCA	
			P: CGCTGGAGGTGCAAAGGACA	
*tgf-*β	*Danio rerio*	AY178450	F: TGCGCAAGCTTTACATTGAC	93
			R: AGGACCCCATGCAGTAGTTG	
			P: TGGATCCACAAGCCCAAGGG	
*tnf-*α	*Danio rerio*	AY427649	F: GCGCTTTTCTGAATCCTACG	169
			R: AAGTGCTGTGGTCGTGTCTG	
			P: TGCACGCAGGAGCCTGAATC	
*reca*	*V. anguillarum*	LC370212.1	F: ATCGCGGCTCCCTTTAAACA	248
			R: AGAGAATCCAGCCGCCGCCATGG	
			P: AACTCGGCTGGATTGAGCAG	

#### Water eDNA

The qPCR analysis of water eDNA relative abundance of *V. anguillarum* was conducted with the recombination-repair protein A (*reca*) as the gene target. The absolute quantification method was employed to evaluate the level of expression of *reca* of the infected tanks compared to the non-infected tanks.

### Statistics

#### Immune gene expression analysis

In the study, all statistical analysis were performed with the GraphPad Prism software (version = 10.1.1). In the gene expression analysis in gills, the internal calibrator consisted of an average of two housekeeping genes. Three housekeeping genes (*rpl13a, elf1α*, and *β*-actin) were tested with NormFinder [17] and the most stable combination (i.e. *β*-actin and *elf1α*) was used to perform the calculations in this study. For each gene, timepoint and group, outlier Ct values from qPCR analysis were removed using the GraphPad Outlier calculator (Alpha = 0.05). The datasets were analysed with Shapiro-Wilk normality test to evaluate the normal (Gaussian) distribution. In the case where the datasets were normally distributed, a one-way ANOVA with Tukey's multiple comparisons test was conducted. Alternatively, a non-parametric Kruskal-Wallis test with multiple comparisons was performed. The evaluation of immune gene regulation between the two fish densities was conducted by comparing the ΔΔCt with either a student-s *t*-test for parametric datasets or Mann-Whitney for non-parametric datasets.

#### Correlation analysis

A correlation analysis was performed between Ct values of *V. anguillarum* eDNA and immune gene expression of zebrafish. The normality of the datasets was assessed as described for the gene expression analysis. The average Ct values from the eDNA relative abundance of *V. anguillarum* were correlated to fish immune gene ΔΔCt values. In the case where the datasets were normally distributed, a Pearson test was conducted. Alternatively, a non-parametric Spearman test was performed. For all analyses, the minimum level of significance was settled at *p* < 0.05.

#### Ethical statement

Experimental procedures were performed according to a license issued by the Experimental Animal Inspectorate, Ministry of Environment and Food, Denmark with license number 2021–15–0201–00,951.

## Results

### Clinical Signs

**Fig. 1:**
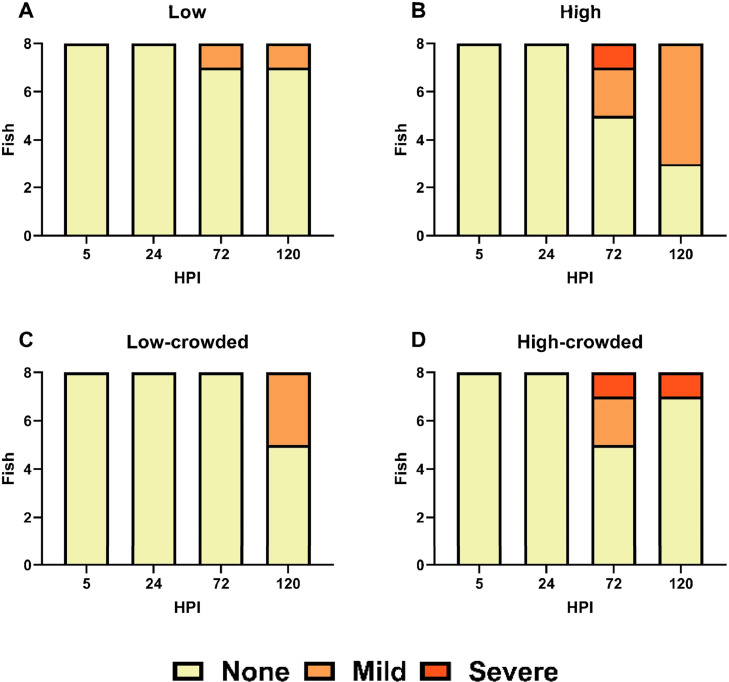
Clinical Signs assessment performed concurrently with fish sampling all along the experimental infection of zebrafish with *V. anguillarum*. For each sampling point, fish (*n* = 8 per group) Clinical Signs were assigned to either none, mild or severe. HPI: hours post infection.

The Clinical Signs monitored throughout the experiment displayed different patterns despite the limited sampling size (*n* = 8 fish/tank). Within the low fish group (Fig. 1A), mild Clinical Signs were observed at 72 and 120 HPI. However, in the low-crowded fish group, mild Clinical Signs were recorded at 120 HPI in a larger proportion (Fig. 1C). Regarding the high bacterial concentration groups, no differences between the two fish density was noticeable until 120 HPI. In the high fish group (Fig. 1B), most sampled fish presented mild Clinical Signs whereas in the high-crowded fish group (Fig. 1D), a single individual showed severe Clinical Signs while the other sampled fish were not showing any Clinical Signs.

### *V anguillarum* eDNA and mortality analysis

**Fig. 2:**
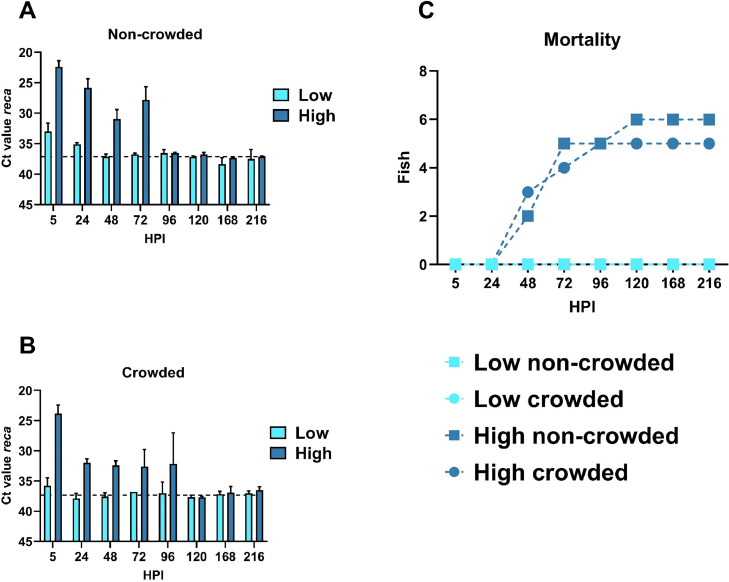
eDNA relative abundance of *V. anguillarum* (detected with the gene *reca*) from water sampling in non-crowded (A) and crowded (B) experimental conditions. The dotted line represents the average Ct values in the control groups for non-crowded and crowded conditions. Mortalities recorded in the different experimental groups (*n* = 40 fish per group) (C). HPI: hours post infection.

The level of *V. anguillarum* eDNA was monitored through water sampling all along the experiment (Fig. 2A and 2B). Among all the filtered samples, the concentration of eDNA varied from 21 to 529 ng/µL. In Fig. 2A and 2B, the dotted line indicates the average Ct values in the mock-infected control groups for non-crowded and crowded conditions. In the low bacterial concentration groups, *V. anguillarum* was detected at 5 and 24 HPI for the low fish group and exclusively at 5 HPI for the low-crowded fish group. Despite witnessing Clinical Signs, the low bacterial concentration bath challenge was sublethal compared to the high bacterial concentration. In the high bacterial concentration groups, the bacteria were detected to a greater extend with lower Ct values. For both fish densities, the peak of detection occurred at 5 HPI. In the high fish group, the signal decreased continuously from 5 to 48 HPI. From 48 to 72 HPI, the signal showed an increase before fading from 96 to 216 HPI. In the high-crowded fish group, the pattern of detection differs from the high fish group. Between 5 and 24 HPI, the signal intensity dropped dramatically. Nevertheless, the signal tends to stabilize from 24 to 96 HPI. From 120 to 216 HPI, the signal in the high-crowded fish group ultimately faded. The mortalities (Fig. 2C) recorded for both high and high-crowded fish densities were overall similar (*n* = 6 and *n* = 5 respectively). For both conditions, most of the mortalities arise at 48 and 72 HPI.

### Immune gene expression

**Fig. 3:**
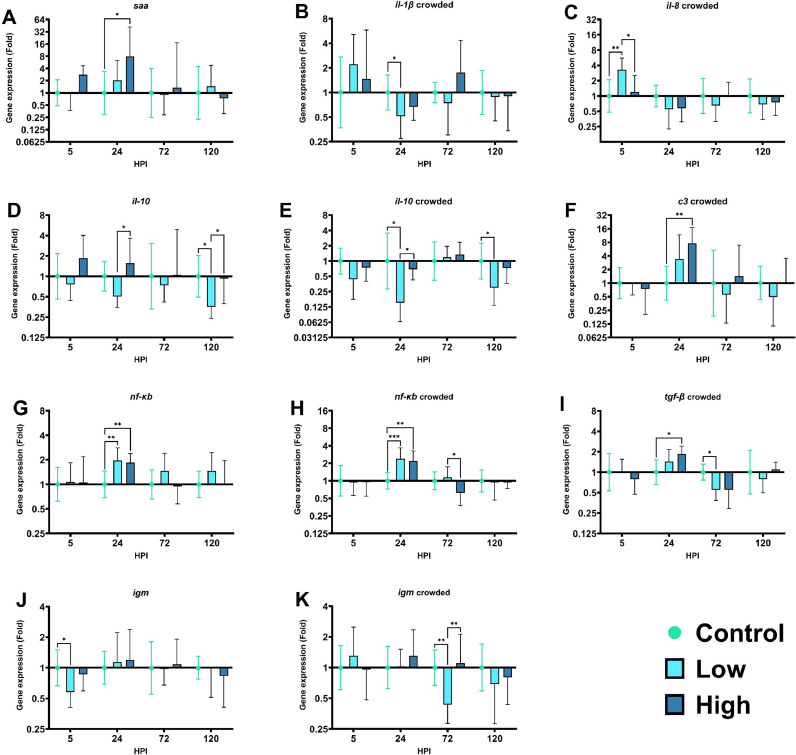
Gene expression analysis of immune relevant genes from zebrafish gills. Only significant results are represented. HPI: hours post infection * *p* < 0.05. ** *p* < 0.01. *** *p* < 0.001.

Among the 9 immune relevant genes investigated in this study (*c3, il-1β, il-8, il-10, nf-κb, saa, tgf-β, tnf-α*), statistically significant differences of immune gene expression were observed predominantly in 7 genes of the crowded fish groups and 4 genes in the non-crowded fish groups, including both high and low bacterial concentration (Fig. 3).

### Immune gene expression in non-crowded fish

In the non-crowded group and high bacterial concentration, the serum amyloid A (*saa*) (Fig. 3A) is the only gene significantly upregulated at 24 HPI.

### Immune gene expression in crowded fish

In the crowded groups, a significant downregulation of *il-1β* (Fig. 3B) at 24 HPI for the low bacterial concentration group was observed. The pro-inflammatory cytokine *il-8* (Fig. 3C), also known as *cxcl8*, was upregulated in the low bacterial concentration group at 5 HPI. The complement component 3 (*c3*) was significantly upregulated at 24 HPI in the high bacterial concentration group (Fig. 3F). The multifunctional cytokine *tgf-β* (Fig. 3I) was upregulated at 24 HPI in the high bacterial concentration group and downregulated in the low bacterial concentration group at 72 HPI.

### Immune gene expression in crowded and non-crowded fish

In both non-crowded and crowded groups, three genes were significantly regulated (*il-10, nf-κb, igm*). The interleukin 10 (Fig. 3D and 3E) showed a comparable pattern for the low bacterial concentration groups. At 120 HPI, *il-10* was downregulated in low and low-crowded fish groups. In addition, another downregulation was observed at 24 HPI in the low-crowded fish group. In the high bacterial concentration groups, at 24 HPI the gene shows an upregulation compared to the low bacterial concentration for both fish density. Regarding the gene expression of the nuclear factor κB (*nf-κb*) (Fig. 3G and 3H), there were no major differences for both fish densities and infective concentration at 24 HPI. However, the gene was downregulated in the high-crowded fish group at 72 HPI compared to the low-crowded fish group. Finally, *igm* (Fig. 3J and 3K) was downregulated at 5 HPI in the low fish group and 72 HPI in the low-crowded fish group.

### Effects of fish density on immune gene expression

**Fig. 4:**
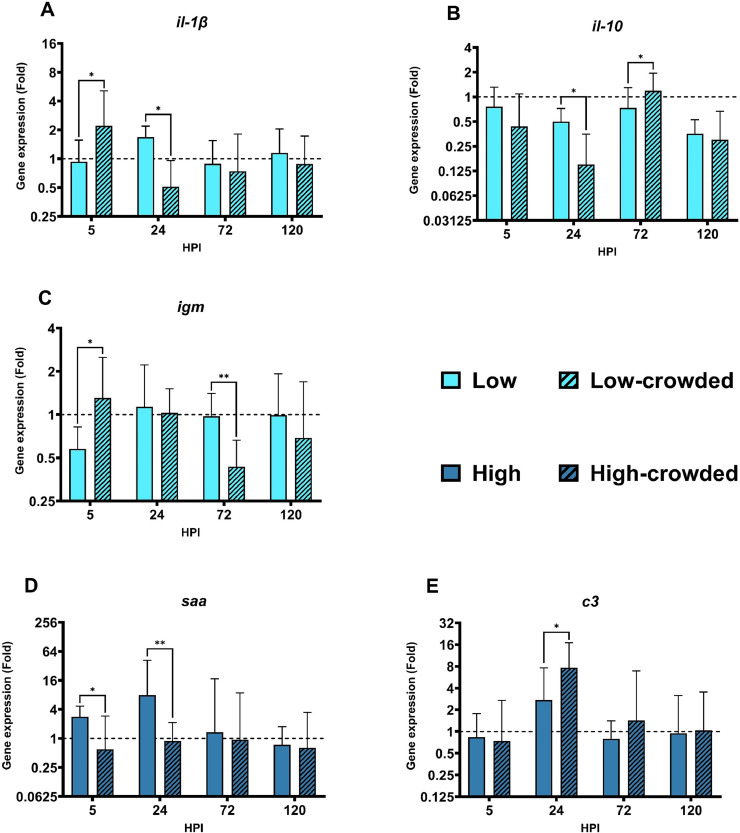
Differential immune gene expression from zebrafish gills according to fish densities. Presented results show statistical significance for both low and high dose of infection. HPI: hours post infection * *p* < 0.05. ** *p* < 0.01.

In Fig. 4, the immune gene regulation (calculated with their respective control) of low/low-crowded and high/high-crowded were compared. Significant differences in gene regulation between fish densities have been noted for three genes (*il-1β, il-10, igm*) in low bacterial concentration and two (*saa, c3*) under high bacterial concentration. In the low bacterial concentration groups, *il-1β* regulation (Fig. 4A) differs between the two fish densities in the early stage of infection (5 and 24 HPI). Regarding *il-10* (Fig. 4B), divergences in gene expression were noted among the two fish densities at 24 et 72 HPI. In the case of *igm* (Fig. 4C) the gene regulation was altered at 5 and 72 HPI. In the high bacterial concentration, *saa* (Fig. 4D) was differentially regulated between the two fish stocking conditions at 5 and 24 HPI. Lastly, the gene *c3* (Fig. 4E) was significantly upregulated to a higher extent in the crowded fish density at 24 HPI compared to the non-crowded fish density.

### Correlation analysis

**Fig. 5:**
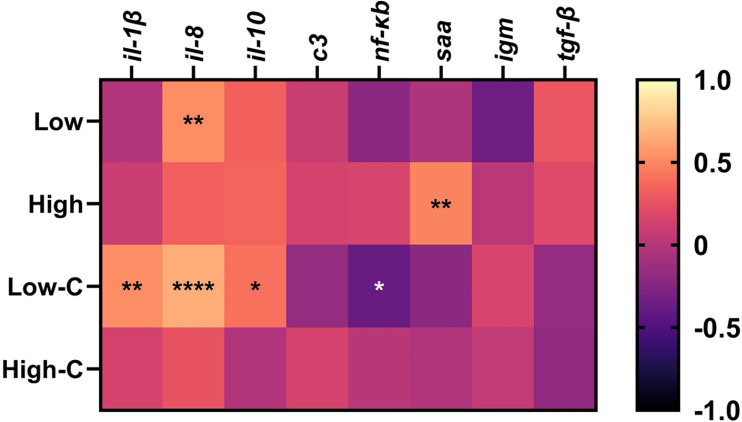
Correlation matrix of zebrafish gills immune gene expression depending on infection pressure (low and high) and fish densities. C: crowded. * *p* < 0.05. ** *p* < 0.01. *** *p* < 0.001. **** *p* < 0.0001.

The correlation analysis (Fig. 5) has demonstrated significant results for all conditions except for the high-crowded fish group. The most abundant correlations (*n* = 4) were found in the low-crowded fish group. The gene that correlated the most to *reca* from *V. anguillarum* was *il-8* with an observed correlation for both fish density groups with the low bacterial infection concentration. In the non-crowded fish groups, *il-8* was positively correlated with low bacterial concentration and *saa* with high bacterial concentration. In the low-crowded fish group, *il-1β, il-8* and *il-10* were all positively correlated whereas, *nf-κb* was negatively correlated.

## Discussion

In the present study, water environmental DNA was collected throughout a waterborne infection model, involving zebrafish and infected with *V. anguillarum* to describe the pattern of pathogen detection in the environment related to disease progression (immune gene expression, Clinical Signs, mortality) in fish. Two different fish densities and bacterial concentrations for non-lethal and lethal infection were used to delimit water eDNA relative abundance when morbidity and mortality began and to elucidate the effect of crowding.

### Zebrafish immune response against *V. anguillarum*

In our experiment, the most significant result involved the expression of *nf-κb*. The transcription factor *nf-κb* is known to play an important role notably in the regulation of immune responses and in the inflammatory process [18]. In teleost fish, the gills are an essential organ, constantly exposed to infections by water-borne microorganisms. The gill-associated lymphoid tissues (GIALT) are widespread among fish and, play a significant role to contain infections caused by pathogens [19]. Therefore, in the scope of vibriosis, several studies have investigated the immune reactions in different fish models. Gills transcriptomic analysis after *V. anguillarum* exposure have been performed in rainbow trout (*Oncorhynchus mykiss*) and the in turbot (*Scolphthalmus maximus*). In rainbow trout, most of the enriched pathways were involved in immune and inflammatory response of including the NF-κB signalling pathway. On the other hand, it was reported in turbot that, the protein interactions involved in TGF-β signalling pathway, the production of inflammatory factors, and endocytosis regulation were most significantly enriched [20,21]. In addition, immune gene expression studies after *V. anguillarum* challenge have been conducted in Japanese flounder (*Paralichthys olivaceus*). In fish exposed to live *V. anguillarum* gene expression of *tnf-α* and *nf-κb* in muscle culminated at 48 HPI [18]. In Japanese flounder exposed to a *V. anguillarum* bacterin, several cytokines involved in the inflammatory process (i.e. *il-1β, il-6, tnf-α, il-8r*) were upregulated in gills three days post exposure [22]. In zebrafish larvae, *nf-κb* mediates inflammation and the recruitment of neutrophils when exposed to bacterial lipopolysaccharide and the pathogenic bacteria *Edwardsiella piscicida* [23,24], which agreed with our findings. Another result congruent with previous infection studies with *V. anguillarum* is the expression of *tgf-β* that was found to be significantly upregulated in high-crowded fish group. In zebrafish, this cytokine is involved in host tolerance and regulation of both innate and adaptive immunity [25]. In this study inflammation was indicated by up-regulation of the cytokine (*il-8*) and different expression profiles of *nf-κb* and *tgf-β* in zebrafish gills. In zebrafish, the inflammation process is important in the mediation of innate immune response [26]. From the previous studies cited above, the inflammatory process seems to be a major component of the immune response against *V. anguillarum*.

### Effect of crowding on the zebrafish immune response

In the present experiment, the impact of crowding on the zebrafish immune gene expression was investigated with a *V. anguillarum* bath challenge. According to the bacterial concentrations (i.e. low or high), genes differed between crowding densities. In fish exposed to the low bacterial concentration, *il-1β, il-10* and, *igm* were differentially regulated. The effect of crowding in fish has been mainly investigated in aquaculture related species. In rainbow trout reared for thirty days under different densities (10, 40, 80 kgm^3^), it was demonstrated that the rearing conditions inducing a significant downregulation of *tnf-α, il1-β* and *il-8* in head-kidney in the two highest fish densities [27]. Similarly, in turbot placed in high density (19,1 kgm^2^), the immune response in skin also resulted in downregulation of *il1-β* and *tnf-α* [28]. However, the crowding effect on immune gene expression seems to be species dependent. For example, contrary to the last two examples, high stocking density in fine flounder (*Paralichthys adspersus*), induced an upregulation of immune-related genes (*il1-β, il-8, tnf-α*) in head kidney and an increased stocking density in grass carp (*Ctenopharyngodon idella*) led to splenic tissue damages and significant upregulation of inflammatory genes (*il1-β* and *tnf-α*) [29,30]. Surprisingly, *il-1β* was not found to be upregulated despite the cytokine plays a major role the in mediation of innate immune response, especially against bacterial infection [31]. In our study, *il-10* and *igm* were downregulated at different sampling points. In zebrafish, *il-10* is essential for gills homeostasis and anti-inflammatory function and *igm* plays an important role in the humoral immunity of teleost fish [[32], [33], [34]]. The downregulation of these immune genes follows results described in rainbow trout and turbot indicating a conserved effect of crowding among these fish species. It is reasonable to speculate here that the infection pressure of the low bacterial concentration was not sufficient to compensate for the crowding effect which seems to induce a downregulation of immune gene in zebrafish. In addition, this idea is reinforced by the fact that the downregulation of *il-1β* and *il-10* was not observed in the high and high-crowded fish groups. Nonetheless, the downregulation of *igm* seems related to the bacterial concentration and the underlying mechanism explaining the observed downregulation remained unclear. In the high bacterial concentration, *saa* and *c3* were found to be differentially expressed. In fish, the serum amyloid A plays a role in the suppression of both bacteriolytic activity and inflammatory signals of neutrophils and on the other hand, promotes their mobilisation towards wounds [35]. In the present experiment, *saa* is significantly upregulated only in the high fish group at 24 HPI, indicating antimicrobial activity. The downregulatory effect of crowding may therefore have suppressed the regulation of *saa* in the high-crowded fish group. In zebrafish eggs, the complement system, in combination with lysozyme are major components of bacteriolytic mechanisms [34]. Furthermore, in air-exposed adult zebrafish, the *c3* gene expression in liver was upregulated at 24 h post stress [36]. Our results of *c3* gene expression in high-crowded zebrafish gills share the same trends as observed following air-exposure. Nevertheless, despite the observed up- and downregulations induced by crowding, the mortalities were overall similar between high and high-crowded fish groups, suggesting a minor impact of crowding over the course of the disease.

### Detection of *V. anguillarum* eDNA

The detection of *V. anguillarum* eDNA in the tank water was acknowledged for both bacterial infection concentrations and fish densities. Previous studies described that physicochemical parameters of the water influence the decay rate of eDNA. A study investigating the effect of the temperature on the detection of *Fenneropenaeus chinensis* water eDNA [37]. The results indicated that within 8 h, the number of copies per mL of *F. chinensis* mitochondrial cytochrome I (COI) gene at 10 °C and 25 °C shifted respectively from 682.56 ± 43.15 and 614.87 ± 78.88 to 627.12 ± 34.96 and 33.33 ± 31.34. The effect of pH on eDNA degradation rates was explored in another study. The results demonstrated that the frame of detection of COI from *Daphnia pulex* was longer in acidic conditions (pH 4) compared to neutral (pH 7) and alkaline (pH 10) conditions [38]. Considering our experimental conditions (pH 7.5; 27 °C), the decay rate of the eDNA from *V. anguillarum* was high and the detection profile especially in Low fish groups illustrated this fact. The initial bacterial concentration in Low dose (2.15 × 10^5^ CFU/mL), the time of exposure (60 s) and the sampling did not allow the fish bacterial burden to increase enough to extend the signal originating from bacterial shedding from *V. anguillarum* in the water and therefore, accelerated the signal fading. Nonetheless, the detection of *V. anguillarum* eDNA in High fish groups for an extended period confirmed the successful colonisation of the host by pathogen and the ongoing vibriosis.

In conclusion, this study indicates that water eDNA is a suitable bioindicator to monitor vibriosis in zebrafish. The profile of *V. anguillarum* eDNA also correlated with gene expression of *il-1β, il-8, il-10, nf-κb* and *saa* in zebrafish gills and coincided with Clinical Signs observed on the fish. Therefore, it is hereby suggested that eDNA in the water is a valid marker of disease status in fish using this model. Furthermore, the present results confirmed that mild stress induced by crowding had an impact on the expression of immune-related genes in both low (*il-1β, il-10* and *igm*) and high (*saa* and *c3*) bacterial concentrations. A difference in eDNA relative abundance was demonstrated between a non-lethal and a lethal exposure. Further studies should investigate different fish pathogens (e.g. parasites, fungi) with regard to eDNA profiling in the course of infection performed under

## CRediT authorship contribution statement

**Cyril Henard:** Writing – review & editing, Writing – original draft, Visualization, Methodology, Investigation, Data curation. **Manuel Blonç:** Writing – review & editing, Investigation. **Hanxi Li:** Writing – review & editing, Investigation. **Yajiao Duan:** Writing – review & editing, Investigation. **Louise von Gersdorff Jørgensen:** Writing – review & editing, Supervision, Conceptualization.

## Declaration of competing interest

The authors declare that they have no known competing financial interests or personal relationships that could have appeared to influence the work reported in this paper.

## Data Availability

Data will be made available on request.
